# Supplementary material for: Improvement of Cell Culture Methods for the Successful Generation of Human Keratinocyte Primary Cell Cultures Using EGF-Loaded Nanostructured Lipid Carriers
Source: Biomedicines. 2021 Nov 6;9(11):1634. doi: 10.3390/biomedicines9111634 (PMC8615600; doi:10.3390/biomedicines9111634)
Supplement: Supplementary file 1 [file biomedicines-09-01634-s001.zip › biomedicines-1429215-supplementary.pdf]

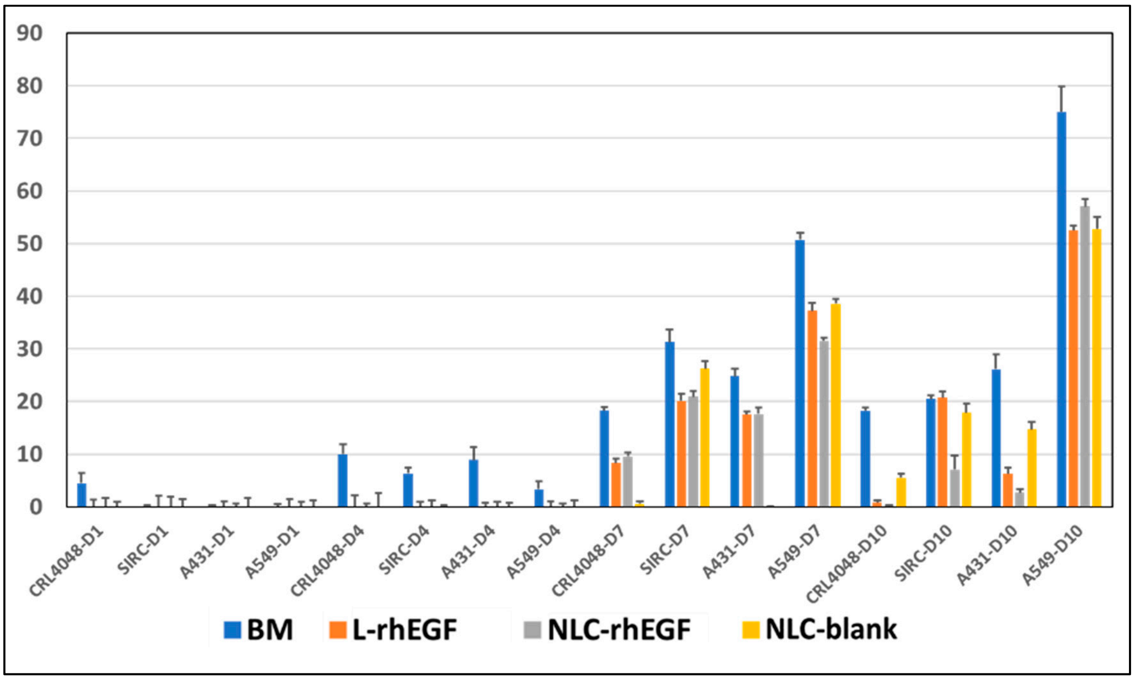

Supplementary Figure S1. Biosafety analysis. DNA released by different cell types cultured in the different media used in this work. CRL4048, SIRC, A431 and A549 were quantified after 1, 4, 7 and 10 days of follow-up (D1, D4, D7 and D10, respectively). Values are averages with error bars showing standard deviations.
